# Hints to Diagnose Intracranial Rosai-Dorfman Disease: A Case Report and Literature Review of Cases in Saudi Arabia

**DOI:** 10.7759/cureus.51204

**Published:** 2023-12-27

**Authors:** Lina M Asiri, Abdulaziz M Alghamdi, Turki Alzidani, Riadh Rebai

**Affiliations:** 1 College of Medicine, Qassim University, Buraydah, SAU; 2 College of Medicine, King Saud Bin Abdulaziz University for Health Sciences College of Medicine, Jeddah, SAU; 3 Neurological Surgery, King Faisal Medical Complex, Taif, SAU; 4 Neurological Surgery, King Fahad General Hospital, Jeddah, SAU

**Keywords:** meningioma, rosai-dorfman disease, central nervous system, extranodal sinus histiocytosis with massive lymphadenopathy, case report

## Abstract

Rosai-Dorfman disease (RDD) was recognized as a distinct clinical entity by Rosai and Dorfman in 1969. It is a rare histiocytic proliferative disorder that occurs in various locations and occasionally involves the central nervous system. In this article, we aim to describe a case of intracranial RDD and to provide a review of the literature on intracranial RDD in Saudi Arabia. A 37-year-old woman presented with a history of generalized seizures. Physical examination disclosed bilateral cervical lymphadenopathy with no neurological deficit. Brain magnetic resonance imaging (MRI) demonstrated an extra-axial, homogenous, Gadolinium-enhancing, space-occupying lesion with extensive dural involvement. The patient was successfully treated by total surgical resection. Postoperatively, the patient did not receive any adjuvant therapy. Biopsy with immunohistochemical analysis confirmed the diagnosis of intracranial RDD. On follow-up examination, six months later, there was no recurrence of the lesion.

A preoperative diagnosis of intracranial RDD is challenging since its MRI appearance can be similar to other intracranial diseases. Herein, we discussed some neuroradiographic findings that might help distinguish RDD from other intracranial diseases.

## Introduction

Rosai-Dorfman disease (RDD), also known as sinus histiocytosis with massive lymphadenopathy (SHML), was first described by Rosai and Dorfman in 1969 [1]. RDD is a rare idiopathic histiocytic proliferative disorder, with a prevalence of 1:200000 [2]. It generally presents with bilateral massive but painless cervical lymphadenopathy. Other accompanying features, such as fever, neutrophilia, elevated erythrocyte sedimentation rate (ESR), and polyclonal hypergammaglobulinemia, may be found in RDD patients [1]. Interestingly, the disease can affect any system, with the most common extranodal sites being the skin, orbit, upper respiratory tract, and bone, which has been reported in up to 43% of cases [3].

In intracranial RDD, the most involved structures are the suprasellar region, cerebral convexities, cavernous sinuses, parasagittal region, and petroclival region. Infratentorial parenchymal lesions are considered the most frequent [4]. The clinical manifestation of intracranial RDD varies widely, depending on the size, exact location, and number of lesions. The most frequent symptoms include headache, seizures, and cranial nerve deficits [3]. The typical radiological findings of RDD with central nervous system involvement (CNS-RDD) show an extra-axial dural-based enhancing mass with perilesional vasogenic edema [3,4]. Classically, histopathological examination is characterized by marked expansion of the lymphatic sinuses with emperipolesis, a distinctive feature whereby well-preserved lymphocytes are engulfed by histiocytes [5]. Although CNS-RDD is extremely rare, occurring in less than 5% of all cases of RDD, it represents a significant diagnostic challenge since it closely simulates meningioma both neurologically and especially radiographically [5,6].

In this article, we present a rare case of intracranial dural-base RDD, with concurrent clinical lymph node involvement, and a review of the literature of similar cases in reported Saudi Arabia.

## Case presentation

**Figure 1:**
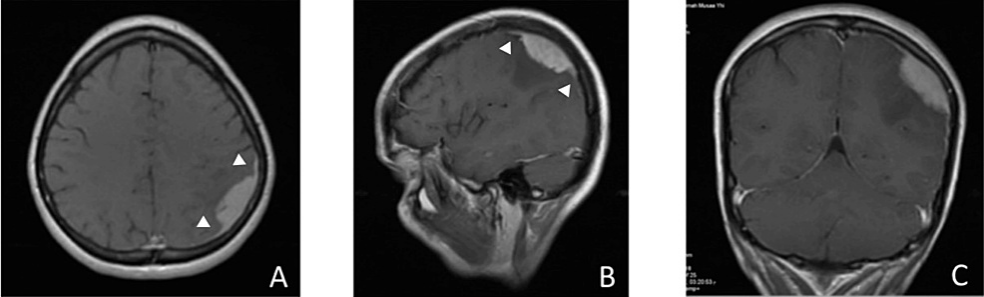
Preoperative magnetic resonance imaging (MRI) Axial (A), sagittal (B), and coronal (C) views of T1-weighted brain MRI show a homogeneously enhancing lesion surrounded by edema in the left parietal region. Note the dural tail sign (white arrowhead).

A 37-year-old female patient presented to the hospital with recurrent episodes of generalized epileptic seizures over two months. The seizure started with rigid extensions of the limbs and trunk lasting for 10 seconds, followed by a rhythmic contraction of the arms and legs. The patient experienced loss of consciousness and autonomic signs during the attack. The postictal phase was marked by confusion that lasted several minutes after the seizure episode. She had no chronic medical conditions and no surgeries. Differential diagnoses were vascular, e.g. subarachnoid hemorrhage (SAH), infection, e.g., brain abscess, and neoplasms, e.g., extra-axial or intra-axial brain tumors. On physical examination, the patient was conscious, and a bilateral non-inflammatory adenopathy at the anterior border of the sternocleidomastoid muscle was observed. Further examination revealed no focal neurological deﬁcit. The patient had a computed tomography (CT) scan of the brain. We saw the report and there were no scans available. Magnetic resonance imaging (MRI) of the brain (Figure 1) demonstrated an extra-axial, homogenous, Gadolinium-enhancing, space-occupying lesion in the left parietal convexity region with extensive dural involvement.

**Figure 2:**
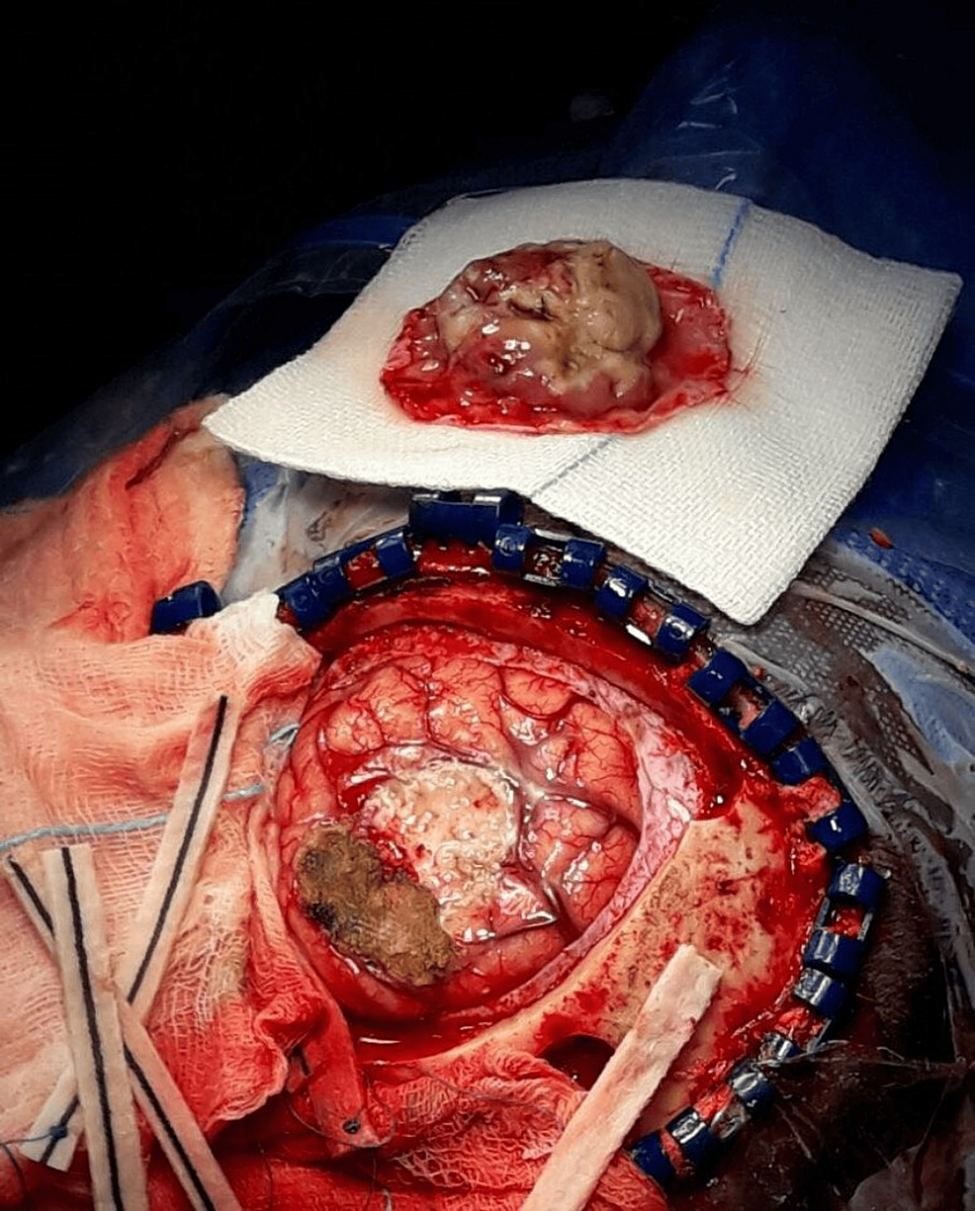
Intraoperative photograph Pale yellow resected mass with dural attachment

Based on the MRI findings, the diagnosis prior to surgery was meningioma. Computed tomography (CT) scans of the neck and abdomen were unremarkable. The primary indication for surgery was the recurrent convulsions. Preoperatively, anticonvulsants and a short course of steroids were initiated. Subsequently, the patient underwent parieto-occipital craniotomy with Simpson grade I en-bloc total excision of the lesion with the dural attachment. Duraplasty with pericranial flap was performed. The lesion was extra-axial, well-defined, xanthochromic, soft, and dural-based, with no invasion of the surrounding tissue (Figure 2).

**Figure 3:**
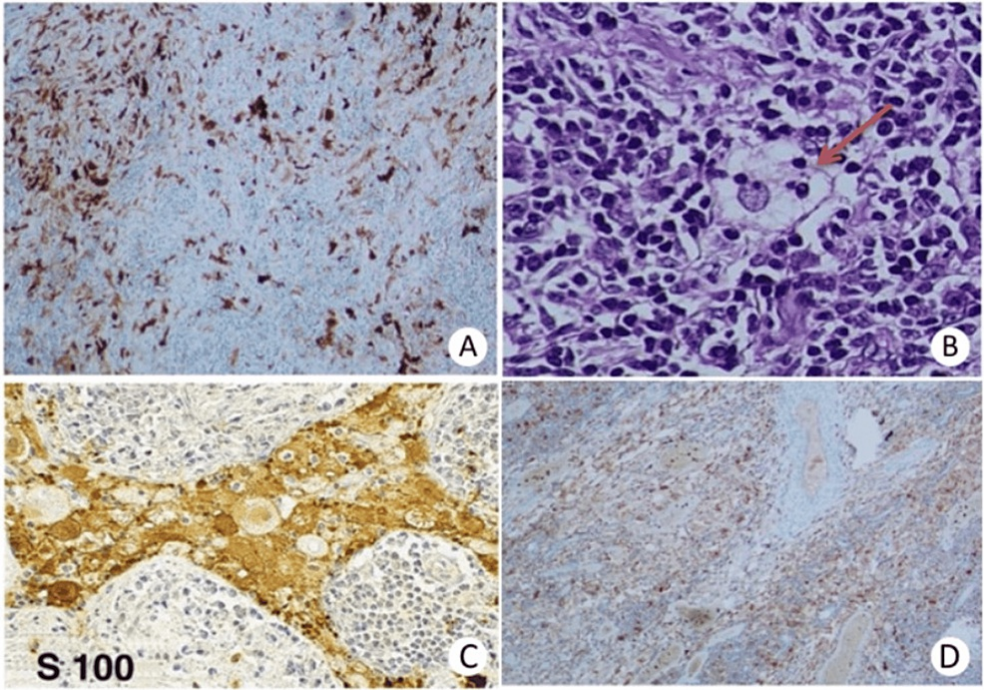
Histopathological findings (A, C) Immunohistochemistry stain shows strong reactivity to S-100. (B) H&E 400x. High power view of extra-nodal RDD showing large pale histiocytes with vesicular nucleus demonstrating emperipolesis (ingested lymphocyte in the cytoplasm of histiocyte). (D) The histiocytic population is highlighted by stains for CD68. RDD: Rosai-Dorfman disease; H&E: hematoxylin and eosin

Histopathological examination of the mass confirmed histiocytosis displaying emperipolesis (lymphophagocytosis). Moreover, the immunohistochemical report revealed that proliferating histiocytes showed strong positivity for cytoplasmic S100 and CD68 protein, but negative expression of CD1a, features that support the diagnosis of RDD (Figure 3).

**Figure 4:**
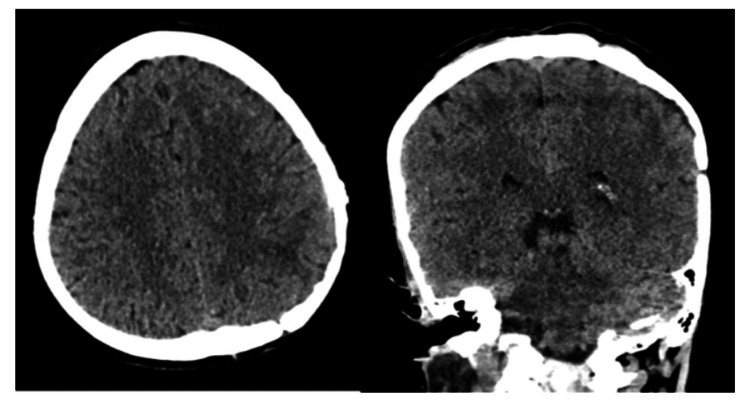
Computed tomography (CT) scan of the brain Six-month postoperative follow-up images confirmed no recurrence.

After surgery, the patient recovered uneventfully and was observed in the recovery room for two hours, shifted to the inpatient ward, stayed there for five days, was followed clinically, and then was discharged home on anticonvulsants and in good health. On regular follow-up, incomplete regression of her cervical adenopathies was noticed. Six months later, the patient remained asymptomatic, and the latest CT brain revealed no evidence of recurrence (Figure 4).

## Discussion

RDD is an uncommon reactive histiocytic proliferative disorder of dubious etiology [1]. Despite the progress made in the understanding of the biology of Langerhans cell histiocytosis (LCH) and Erdheim-Chester disease (ECD), the knowledge regarding the pathogenesis of RDD has lagged [7]. Nonetheless, some studies proposed that immune-system dysregulation caused by specific infections, such as human herpes virus type 6 (HHV-6) and Epstein-Barr virus (EBV), may be implicated in the etiology of RDD [3]. Other studies investigated the molecular profile of the disease; Richardson et al. confirmed the presence of the BRAF mutation in a single case of CNS-RDD [8]. Recently, mutually exclusive KRAS and MAP2K1 mutations were identified in up to 33% of RDD cases, supporting the idea that in at least a subset of cases, the disease is a clonal process [7].

Intracranial RDD predominantly affects males with a male-to-female ratio of 1.8:1.0; it has been reported in all age groups with a mean age of 39 years old [9]. According to the most updated review, approximately 299 cases of RDD with CNS involvement were reported in the literature. Overall, 78.3% (234/299) of CNS-RDD occur intracranially while 13.7% (41/299) involve the spine, and 8.0% (24/299) have mixed intracranial and spinal lesions simultaneously [4,9]. We searched the English literature using the MeSH tool from the PubMed database, and we used the keywords “Rosai-Dorfman disease, sinus histiocytosis with massive lymphadenopathy, and central nervous system”. A literature review of similar cases reported in Saudi Arabia revealed a total of six cases in the literature. Their details are shown in Table 1 [10,11].

**Table 1:** Synopsis of the reported cases of intracranial Rosai-Dorfman disease in Saudi Arabia Central nervous system: CNS, Not mentioned: NM, Relative afferent pupillary defect: RAPD, None other specified: NOS

Case number	Author, year	Gender	Age, in years	Symptoms	Location of the CNS involvement	Relevant systemic Involvement	Management/intervention	Follow-up period	Recurrence
1	Jaudah A. Al-Maghrabi [10], 2022	Female	24	NM	Optic chiasm	None	Surgical biopsy	NA	NM
2	Jaudah A. Al-Maghrabi [10], 2022	Female	44	NM	Ipsilateral temporal lobe	None	Surgical resection	14 months	None
3	Jaudah A. Al-Maghrabi [10], 2022	Male	39	Seizures	Ipsilateral parietal dura and subcortical white matter	Ipsilateral parietal bone	Surgical resection	12 months	None
4	Tariq A. Alzahem [11], 2021	Male	NM	Decreased vision, left lower eyelid swelling, proptosis, and RAPD	Brain mass, NOS	facial masses, bilateral paranasal sinus infiltration, and bilateral Intraconal and extraconal ocular masses with right intra-canalicular optic nerve compression	Management of the CNS involvement is NM, oral prednisolone, chemotherapy, and debulking of the left orbit’s masses were done for the ophthalmological symptoms	NM	NM
5	Tariq A. Alzahem [11], 2021	Female	70–74.9	Right upper eyelid swelling with enlargement of right lacrimal gland causing dystopia	Intracranial and dural masses, NOS	Right superotemporal and left inferotemporal extra-conalocular masses, and enlargement of the V2 and V3 branches of the trigeminal nerve	Management of the CNS involvement is NM, oral prednisone was given for the ophthalmological symptoms	NM	NM
6	Tariq A. Alzahem [11], 2021	Female	NM	Right lower eyelid swelling, proptosis, and mild limitation of extraocular muscles	Brain mass extension from a bilateral maxillary sinus infiltration	Inferior extraconal ocular mass	Management of the CNS involvement is NM, radiotherapy, and oral prednisolone were done for the ophthalmological symptoms	NM	NM

Neuroimaging classically shows an extra-parenchymal homogeneously enhancing lesion, surrounded by edema with coexisting irregular thickening of the adjacent meninges. Dural-based RDD lacks specific radiographic features; accordingly, it is usually misdiagnosed as meningioma [3,4]. However, recently, many articles have reported distinctive radiological features that raise suspicion of CNS-RDD. Zhu H et al. believed that a typical representation of isointense masses with a strong intralesional hyperintensity on T2-weighted or fluid-attenuated inversion recovery (FLAIR images irrelevant to calcification can suggest the diagnosis of RDD [12]. Besides, the absence of hyperostosis, bony erosion, or calcification should suggest RDD as a differential diagnosis of meningioma. Other important radiological characteristics of CNS-RDD are the lobulation signs that are seen at the margin of the lesion, and the irregularly thickened meningeal extension to the brain parenchyma “pseudopodium” [9]. In contrast, an intra-tumoral vascular blush from branches of the external carotid artery on digital subtraction angiography (DSA) is pathognomonic for meningioma, making a diagnosis of RDD less likely [13]. With that being said, intraparenchymal CNS-RDD also mimics lymphoma and tuberculous granuloma; therefore, the accurate diagnosis of CNS-RDD is still difficult preoperatively [9].

Microscopic examination shows a fibrous stroma infiltrated by dense inflammatory cells, along with prominent sinusoidal dilatation, resulting in effacement of nodal architecture, particularly in advanced cases [14]. The lymph node sinuses are occupied by a mixed cell population of lymphocytes and histiocytes with a large vesicular nucleus and abundant clear cytoplasm [6]. The majority of histiocytes contain intact lymphocytes and plasma cells within their cytoplasm exhibiting emperipolesis. Although having a great diagnostic significance, this representative feature of RDD may be inconspicuous in intracranial disease [5]. The histopathologic differential diagnoses include inflammatory pseudotumor/plasma cell granuloma (PCG), LCH, and meningioma. Of note, S100 positivity is observed in both LCH and RDD [6]. Yet, LCH can be readily differentiated from RDD by the characteristic indented nucleus, which contains Birbeck granules in addition to the positive CD1a [6]. Meningiomas also have S100+ but are distinguished from RDD by the epithelial membrane antigen (EMA+) marker, whereas PCG is ruled out by the S100- and the absence of emperipolesis [6]. Therefore, histopathological examination and immunohistochemical staining are essential for a definitive diagnosis of RDD [5].

To the best of our knowledge, there are no standardized treatment guidelines for CNS-RDD. Most instances of RDD that involve the CNS are treated with surgical excision, which may be indicated in unifocal or symptomatic disease, it is considered an effective treatment for those with Intracranial RDD [5]. Surgical resection efficacy is not only confined to relieving compression of vital organs but also provides enough specimens for pathological diagnosis [9]. Alternatively, for patients with multifocal irresectable disease, systemic therapy may be required, which includes corticosteroids, chemotherapy, radiotherapy, and immunomodulatory therapy. Notably, adjuvant therapy, such as steroids and chemotherapy, are effective and safe postoperative treatment for relapsing cases or residual lesions [14,15]. However, for those patients with subtotal tumor resection, a low dose of radiation can be beneficial [16].

The prognosis of RDD is excellent with spontaneous remission reported in up to 50% of cases, especially in cutaneous and nodal disease. However, in a few cases, it may recur or may even be fatal. Almost 10% of patients may die as a result of direct complications: infection and amyloidosis [14]. Petzold et al. recommended a five-year (median relapse time) follow-up with brain imaging [16].

The current study has some limitations, as the evidence provided is limited to solitary case reports; however, it is the first study to provide a literature review of intracranial RDD in Saudi Arabia. In general, a broader understanding of the RDD pathogenesis may better guide improved management. More studies are needed to appropriately investigate the genomic landscape of RDD and its therapeutic relevance.

## Conclusions

Intracranial RDD is an uncommon reactive histiocytic proliferative disorder of vague etiology. Up to this date, almost 300 cases of intracranial RDD have been reported in the literature, and our literature review revealed only six reported cases in Saudi Arabia. We reported a surgically managed case of an intracranial RDD that was initially misdiagnosed as meningioma based on radiological features. Intracranial RDD can be often mistakenly diagnosed as meningioma since they both share similar clinical, radiological, and macroscopic features. However, several distinctive radiological features can raise suspicion of intracranial RDD. Nevertheless, intracranial RDD can still mimic other lesions radiologically such as lymphoma and tuberculous granuloma, which makes it more difficult to diagnose it preoperatively. Although a preoperative diagnosis is feasible in many cases, histopathology and immunohistochemical confirmation remain indispensable for a definitive diagnosis. With the absence of a standardized treatment guideline for Intracranial RDD, most cases are treated by surgical excision with an excellent reported prognosis. Intracranial RDD remains a rare pathological entity with its own diagnostic and management challenges.
